# The Neuroimmunology of Multiple Sclerosis: Fictions and Facts

**DOI:** 10.3389/fneur.2021.796378

**Published:** 2022-02-07

**Authors:** Andrew R. Pachner

**Affiliations:** ^1^Dartmouth–Hitchcock Medical Center, Lebanon, NH, United States; ^2^Geisel School of Medicine, Dartmouth College, Hanover, NH, United States

**Keywords:** multiple sclerosis, neuroimmunology, B cell, CXCL13, cytokine, neuroinflammation, cerebrospinal fluid, chemokine

## Abstract

There have been tremendous advances in the neuroimmunology of multiple sclerosis over the past five decades, which have led to improved diagnosis and therapy in the clinic. However, further advances must take into account an understanding of some of the complex issues in the field, particularly an appreciation of “facts” and “fiction.” Not surprisingly given the incredible complexity of both the nervous and immune systems, our understanding of the basic biology of the disease is very incomplete. This lack of understanding has led to many controversies in the field. This review identifies some of these controversies and facts/fictions with relation to the basic neuroimmunology of the disease (cells and molecules), and important clinical issues. Fortunately, the field is in a healthy transition from excessive reliance on animal models to a broader understanding of the disease in humans, which will likely lead to many improved treatments especially of the neurodegeneration in multiple sclerosis (MS).

## Introduction

I saw my first patient with multiple sclerosis (MS) 50 years ago in 1971 as a medical student. The patient was a young woman who presented with acute myelitis. The patient had 3 years previously developed diplopia which was not clearly diagnosed and resolved within a few weeks. A spinal tap was performed which was equivocal for markers of MS. At that time, the technology for cerebrospinal fluid (CSF) oligoclonal band testing with polyacrylamide gels was the standard (1), which is relatively insensitive in the diagnosis of MS. Also, at that time, the first CT scan of the brain had not yet been performed, and NMR, the precursor of MRIs, was used in biophysics research laboratories, not yet in humans. A hot bath test (2) was performed, and an intranuclear ophthalmoplegia was evoked, which was consistent with the diplopia being caused by an MLF lesion and thus confirmed the diagnosis of MS, using Poser's criteria (3). The worsening of MS symptoms with raised body temperature is called Uhthoff's sign, first described in 1890. The only treatment available for MS in 1971 was corticosteroid medication, which was infused, and the patient improved and went home.

Much has changed since then. Diagnostic approaches and criteria have been refined: hot bath tests have been replaced by much better diagnostic testing, including MRI scanning (4) and highly sensitive oligoclonal band (OCB) testing using isoelectric focusing on agarose gels and immunofixation of IgG bands (5), and increasingly sensitive diagnostic criteria have been developed (6, 7). Along with corticosteroids for acute attacks, we now have 23 FDA-approved immunomodulatory/immunosuppressive drugs (ISDs) (8) to dampen neuroinflammation. MS has an estimated prevalence in the US of about 300 per 100,000 (9), and clinicians are increasingly faced with complex issues in the diagnosis and management of these patients.

## The Three Inherent Challenges in Researching the Biology of MS

Unfortunately, there has been less progress in our understanding of the biology of MS, which has manifested as uncertainty and much confusion about the underlying pathological processes in MS of demyelination, inflammation, and neuronal damage. This unfortunate state has been due to primarily three characteristics of MS:

1. minimal access to involved tissue,

2. highly variable clinical course, and

3. tendency to extrapolate over-simplistically from results of research in the rodent model of MS, experimental autoimmune encephalomyelitis (EAE).

In contrast to diseases where biopsies are readily available, CNS tissue is rarely accessed during life in patients with MS, and autopsy tissue often yields end-stage processes in a disease that usually begins when patients are young. Although considerable information has been obtained from brain biopsies in a relatively small group of selected patients with MS for whom this aggressive procedure was indicated (10), findings are controversial (11), and characterization of the pathology is a continual work in progress (12), especially as meningeal inflammation and cortical demyelination are increasingly recognized and studied (13). The disease is highly heterogeneous and there are at least 15 different clinically distinguishable variants of MS (Table 1) (14), some of which may morph into other variants in any one patient. Thus, the predominant neuroimmunology of one variant may be different from another. Finally, partially because of the above challenges, much of the research in the disease has focused not on MS, but rodents mostly mice, EAE. There have been over 14,600 publications on EAE (as of July 1, 2021), despite the general acceptance that it is not a faithful model of the human disease (15); however, there is no doubt that it provides insights into immunology and inflammation in the CNS (16, 17). Although our lack of understanding about the biology of the disease is unfortunate, it is not surprising given the above challenges and given the fact that neuroimmunology research overlaps the two most complex systems in the human body: the nervous and the immune systems.

**Table 1:** The 15 forms of MS.

**Category**	**Form**
**MS-like diagnosis**	
1.	Asymptomatic-MS pathology found at autopsy
2.	Clinically isolated syndrome (CIS)
3.	Radiologically isolated syndrome (RIS)
**Definite MS each with 4 variants according to inflammatory activity or progression of disability (non-progressive/non-active, non-progressive/active, progressive/non-active, progressive/active)**	
4–7.	Relapsing-remitting MS(RRMS)
8–11.	Primary progressive MS(PPMS)
12–15.	Secondary progressive MS(SPMS)

It is thus important to identify the difference between what is KNOWN vs. what is THOUGHT TO BE KNOWN. I would classify the former as FACT and the latter as FICTION, or more precisely NON-FACT, since some non-facts represent not completely unreasonable hypotheses yet to be proven right or wrong. This review will evaluate critical areas of the clinical neuroimmunology of MS, and separate fact from fiction.

## Fiction #1. MS is an Autoimmune Disease

### FACT #1. MS Has a Definite Inflammatory Component, Especially Early in Its Course, but It May Not Be Truly Autoimmune

One of the pioneers in neuroimmunology and EAE in particular, Byron Waksman, was convinced for decades that MS was autoimmune despite lack of firm evidence, but, toward the end of his career, changed his mind. While describing paradigm shifts for the pathogenesis of MS, he wrote: “EAE, the autoimmunity-based animal model, is not seen as inherently superior to (viral) models like Visna or TMEV. One might be right to regard autoimmunity (as a cause for MS) as a paradigm shift that never quite made it!” (18).

Ascribing the adjective “autoimmune” to a disease with confidence requires the demonstration of an autoantigen, a self-molecule, and an abnormal, damage-inducing, cellular, and/or humoral response to that molecule. Some examples of neuroimmunological autoimmune diseases are myasthenia gravis with autoantibodies against the nicotinic acetylcholine receptor (19) and neuromyelitis optica with autoantibodies against aquaporin-4 (20). Thus far, despite decades of research into the pathogenesis of MS, neither an autoantigen nor a damage-inducing humoral or cellular immune response has been convincingly demonstrated. The most prominent immunological abnormality in MS is the presence of elevated immunoglobulin G (IgG) response in the cerebrospinal fluid (CSF), usually measured as oligoclonal IgG bands (21), yet an intensive search for the target of that IgG response has not identified an autoantigen, and the most common cause of CSF OCBs other than MS is viral infections (22). Of course, many inflammatory diseases that are usually considered “autoimmune” in pathogenesis such as rheumatoid arthritis and systemic lupus erythematosus, do not have well-defined pathogenic cellular or humoral immune responses directed against an autoantigen. However, these diseases have a host of characteristics that are consistent with autoimmunity, while MS lacks a convincing array of these characteristics. For MS, it is hard to prove a negative, i.e., absence of evidence does not mean evidence of absence and thus, the autoimmune hypothesis must still be considered possible. In a recent review of MS treatment (23), the authors, who a decade ago would have classified MS as definitely an autoimmune disease, have scaled back their certainty and described MS as “most likely autoimmune-mediated.”

Experimental autoimmune encephalomyelitis (EAE) is the standard model for the “outside-in” hypothesis for the pathogenesis of MS, in which events such as lymphocyte activation outside of the CNS is the initiating event, followed by influx into the CNS of pathogenic cells from the periphery. In contrast, other MS investigators favor an “inside-out” hypothesis, in which the initiating event is some change within the CNS, such as damage to myelin or neuronal components, and subsequent inflammatory events occur as a response to this damage. These two types of pathogenesis are, of course, not mutually exclusive, but the predominance of one hypothesis vs. the other has considerable significance for research directions and possible therapeutic targeting. The arguments for these two contrasting hypotheses are extensive and beyond the scope of this review but have been summarized in multiple recent review articles (24–26).

## Fiction #2. MS is T-Cell Mediated

### FACT #2. The Mechanism of Damage in MS Is Unknown

When I was reviewing grant applications for the National MS Society from 1998 to 2005, many applications began their background section with the statement: “MS is a T-cell mediated autoimmune disease.” The literature is also full of such confident assertions, e.g., “(MS) is characterized histologically by the infiltration of encephalitogenic TH1/TH17-polarized CD4(+) T cells” (27). These dogmatic statements irritate me, since it is not a proven autoimmune disease (28), and, although T cells may be involved in the inflammatory response, there is no conclusive evidence that it is actually T cell-mediated or that T cells are encephalitogenic in human MS.

A pivotal manuscript by Henderson et al. (29) is especially relevant to this issue. They analyzed pathology in early MS lesions, and, by painstakingly accumulating autopsy samples from 11 patients with early MS, addressed one of the primary challenges in MS, #1 above, i.e., minimal access to involved tissue, especially early in the disease. Two of the most studied specimens were from patients with a duration of MS of 18 and 21 days. Their conclusion was that “adaptive immune activity involving T and B cells is conspicuous chiefly in recently demyelinated tissue, which may show signs of oligodendrocyte regeneration. The findings suggest that plaque formation has some basis other than destructive cell-mediated immunity directed against a myelin or oligodendrocyte antigen,” and thus their conclusion that the involvement of T and B cells is a response to some initial unidentified damaging process, rather than the inducer of the early MS lesion.

The emphasis on T-cells as the primary mediator of the neuroinflammation is to a large extent a consequence of extrapolation from the EAE model, challenge #3 above. The history of EAE research is long (30), but the understanding of the immunological basis of the neuroinflammation grew rapidly pari passu with the growth of immunological research after World War II. Methodologies for cloning subpopulations of lymphocytes were developed and in 1981 Ben-Nun et al. (31, 32) demonstrated that T cell lines from MBP-immunized rats transferred into naïve rats completely reproduced the disease. Experiments over the subsequent 40 years in many different types of EAE have expanded upon this pioneering finding. Although some forms of EAE are not T cell-mediated (33), the majority are. EAE can be induced in a variety of animals, not just rodents, including non-human primates (34) but murine EAE is the most commonly studied because of the ready availability of mutant mice. However, even though EAE was from the outset perceived as a model of not MS, but acute disseminated encephalomyelitis (ADEM) (35, 36), a neuro-inflammatory cousin of MS, there has been an oversimplistic extrapolation of T cell mediation to MS. This has occurred despite the fact that the mediating type of T cell in most forms of EAE is the CD4+ helper T cell, while the predominant type of T cell in MS lesions in the CD8+ cytotoxic T cell (37, 38). The dangers of trying to rigorously extrapolate from animal models of MS to the human disease have been recently extensively reviewed (39). Thus, contrary to the situation in most EAE models, it is highly unlikely that the MS is solely mediated by T cells, although that possibility cannot be completely ruled out given the association of MS with the HLA-D region (40) and the exacerbation of MS with checkpoint inhibitors (41).

The dogma of T cell mediation of MS was repeated so often prior to 2008 that the demonstration by Hauser et al. (42) of potent downregulation in relapsing-remitting MS (RRMS) of neuroinflammatory activity by B cell depletion with rituximab, a monoclonal antibody targeting CD20 on the surface of B cells, was a huge eye-opener to the T cell/MS community. This marked amelioration of the neuroinflammation by depletion of B cells has been in marked contrast to the minimal or absent effect of selective T-cell targeted therapies in MS (43).

Some EAE models require B cells (33), but in almost all EAE models, B cells are irrelevant, further bringing into question the relevance of EAE to MS, since B cells are known to be important in MS pathogenesis. Of course, there is constant interaction between B cells and T cells (44) and thus the success of B cell depletion in down regulating inflammation in MS does not rule out some role for T cells in MS pathogenesis. More on the role of B cells in MS is summarized below in FICTION/FACT #4.

## Fiction #3. Viral Models of MS are Irrelevant Because MS is not a Viral Disease

### FACT #3. Not Only Do Viral Models Provide Insight Into Chronic Demyelinating Neuroinflammation, but Also MS May Indeed Represent a Chronic Infection With a Virus or Other Pathogen

The two most studied viral models are those induced by Theiler's murine encephalomyelitis virus (TMEV) and mouse hepatitis virus (MHV). Unlike EAE, where there is a hyperacute neuroinflammatory response causing transient neurological disability with no or stable persistent deficits, genetically susceptible mice infected with TMEV develop progressive weakness over months, associated with demyelination and inflammation with a pathology strongly resembling MS (45). An example of how this model is relevant to MS is in the identification of CXCL13 [Chemokine (C-X-C motif ligand 13)], a B cell-active chemokine, as a strongly upregulated cytokine locally within the CNS both in TMEV infected mice (46, 47) and MS (48, 49). These findings support a plethora of data supporting the critical role of B cells in inflammatory demyelination (summarized below).

The presence of viral models resembling MS has prompted a decades-long search for a viral etiology of MS (50), with the absence of firm evidence for the involvement of any virus (51). Two candidates that have attracted a great deal of interest are Epstein-Barr virus (EBV) and human endogenous retroviruses (HERV). EBV, a large herpes virus whose DNA contains about 170,000 base pairs, very commonly infects humans, with more than 90% of Americans having serological evidence of infection by the time they are adults. The hypothesis that EBV infection plays a role in MS pathogenesis is not recent, with Poskanzer et al. postulating back in 1963 that MS is a late manifestation of an infectious disease common in childhood (52); EBV was not described until 1964, and its link to infectious mononucleosis made by Niederman et al. (53). Multiple studies in the ‘80 s and ‘90 s demonstrated significant increases in anti-EBV antibodies in patients with MS compared to controls, although this finding has not been uniformly consistent. EBV genome has been found in MS brains, with one study demonstrating PCR positivity in 91/101 (90%) MS brains and only 5/21 (24%) of control brains (54). As with EBV antibodies, the findings in detection of EBV DNA or RNA in MS brain has not been consistent with some studies being negative (55). The variability in findings has been attributed, at least in part, to differences in the methods employed (56). A recent study has demonstrated that exosomes from patients with MS express EBV-derived proteins (57). Some positive findings of anti-herpes virus drugs in MS, i.e., acyclovir or valacyclovir, in phase 2 studies have prompted calls for further investigations of antivirals in MS (58). The literature in this area is large; fortunately, the role of EBV in MS pathogenesis has been recently reviewed in a very well-written and thorough manuscript (59).

Recently another group of pathogens has attracted interest: HERV, summarized in part in the publication of two international conferences, one in 2015 (60), and the other in 2017 (61). Over evolutionary time, almost all animals have been infected with retroviruses some of which have become integrated into the genome of the host. HERVs comprise about 8% of human DNA, most of which are not transcriptionally active and have a high susceptibility to mutation. Kremer et al. (62) have found that the envelope protein (ENV) encoded by a member of the HERV-W family may be pathogenic in MS since it both reduces the ability of oligodendrocytes to differentiate, possibly contributing to the failure of remyelination in the CNS and, in addition, promotes microglial-mediated axonal damage (63). Temelimab, also called GNbAC1, is a humanized IgG4 monoclonal antibody that binds HERV-W-ENV and blocks its effects on microglia and oligodendrocyte precursors, was tested in patients with MS, and although it didn't reach the primary endpoint, it appeared to have some helpful effects on MRI measures of neurodegeneration (64).

## Fiction #4. The Immune Response in MS is Dominated by Antigen-Reactive Adaptive Immunity, Particularly CD4+ T Cells

### FACT #4. Activation of the Immune Response in MS Is Likely Highly Diverse With Many Different Subpopulations Involved, Including the Innate Immune Response, B Cells, and CNS Resident Cells

The immune response is sometimes simplistically divided into adaptive and innate immune responses, in which the former is associated with highly antigen-specific receptors on cell surfaces of lymphocytes [e.g., T-cell receptor (TCR) or B-cell receptor (BCR)] and the latter consisting of a mix of cells lacking such receptors, as well as antigen-non-specific molecules such as complement. The summary below of the cellular immunology of MS is very brief and deals with only a small fraction of this large field; a more extensive and excellent review article on this topic has been recently published (65).

### “Innate” Immune Cells-

Given the lack of a clearly pathogenic adaptive immune response in MS, as well as the complexity of the innate immune response, it is not surprising that many components of the innate immune response have been implicated as potentially important in MS. An extensive analysis of this area is beyond the scope of this review, but it is worthwhile to identify some key implicated innate cells and systems. The most prominent among innate cells in this regard are cells of the monocyte/macrophage/microglia lineage, which have recently been extensively reviewed (66, 67). There are at least three different classes of these cells in MS tissue: CNS resident cells [microglia and CNS-associated macrophages (68)] and infiltrating cells of this lineage from the peripheral blood.

Microglia are common cells within the CNS, and for many years were thought to be relatively homogeneous. The realization that they were a heterogeneous population began in 1988 when Hickey and Kimura described a subset called perivascular microglia (69), which are located within the basal lamina of cerebral blood vessels, express MHC II antigens constitutively, and are regularly repopulated by bone marrow-derived precursor cells. In contrast, most populations of microglia are sustained by replication *in situ*. Juxtavascular microglia (70) are a cousin of perivascular microglia, and they also express MHC II, but, unlike perivascular microglia, are located just outside the basal lamina, and do not manifest significant turnover. Other populations of microglia are usually defined not by their location within the CNS but by their shape, i.e., ameboid, ramified, or by expression of the protein, allograft inflammatory factor 1(AIF-1), also called ionized calcium-binding adapter molecule 1(IBA1), identifying reactive microglia. Another important group of molecules expressed in microglia has been termed the “sensome,” which allow these cells to detect changes in their environment (71). Microglia are characterized by extreme plasticity, i.e., being able to change from one phenotype to another in response to environmental conditions. Thus, a ramified microglial cell can become a reactive microglial cell in an inflammatory milieu. Sometimes reactive microglia are also called activated, although that term is misleading since most microglia are almost always activated to some extent.

Reactive microglia are a common finding in MS lesions (72). They can produce toxic free radicals which have been hypothesized as being important in the neurodegeneration in MS (73, 74). In contrast, microglia can also be “homeostatic,” and a loss of P2RY12-positive “homeostatic” microglia has been associated with active lesions, with the restoration of this subpopulation in inactive lesions (75). A highly relevant study was performed by van der Poel et al. (76), who studied microglia isolated from gray matter (occipital lobe) or white matter (corpus callosum) from brains from patients with MS and controls in the Netherlands Brain Bank. Microglia showed a transcriptional profile specific to the region, with higher expression of type-I interferon genes in gray matter and higher expression of NF-kappaB pathway genes in white matter in both normals and patients with MS. In MS, white matter microglia showed increased lipid metabolism gene expression, while gray matter microglia showed increased expression of glycolysis and iron homeostasis genes. In this study the “homeostatic gene” P2RY12 was unaltered in normal-appearing MS tissue, implying preservation of microglia homeostatic functions. Like most studies of MS brains, this work was limited by the use of brains from patients with long-standing MS, with an average disease duration of 28.7 years, despite claiming that they were analyzing “early pathological change.” They justified this claim by stating that 37% of total lesions in these post-mortem brains were active (77). However, the “activity” of lesions in late MS is controversial and others have found much less inflammatory activity in brains from that group of patients (78).

CNS-associated macrophages (CAMs) are a highly heterogeneous group of cells, that are not present within the parenchyma itself, but at the interface of the CNS with the periphery, and are sometimes called border-associated macrophages (BAM) (79). Thus, there are macrophages in the perivascular space (pvMΦ) (80), leptomeninges (mMΦ), and choroid plexus (cpMΦ) (81). Most of the neuroinflammation in MS occurs within the parenchyma as demonstrated by the relatively mild inflammatory profile in the subarachnoid space, i.e., CSF, even in highly active disease. However, it is likely that this group of macrophages has a role in MS, but that role has yet to be defined.

Finally, macrophages infiltrate from the blood in patients with MS, and are sometimes referred to as “monocyte-derived macrophages.” Macrophages have long been known to be important in MS pathology (82, 83), but methodologies to clearly differentiate blood-derived macrophages from intrinsic CNS origin cells in MS are controversial. However, monocyte-derived macrophages have clearly different origins, gene expression, and function compared to the other CNS cells of the monocyte/macrophage/microglia lineage (84). Teasing out the relative contributions of the many subsets within the lineage, e.g., CD16+ positive “non-classical” macrophages (85), will continue to be a major challenge.

Neutrophils, also called polymorphonuclear leukocytes, have also been implicated in the pathogenesis of MS (86, 87). These cells have historically been overlooked in MS research because of the difficulty with working with them in the laboratory and they are being infrequently observed in post-mortem brain tissue. In addition, the neutrophil phenotype can be plastic. However, in some forms of EAE, the involvement of neutrophils is prominent (88, 89), and their depletion inhibits the disease (90). Neutrophils can be detected in the CSF early in the disease and early in relapses (91), so they may be more important in the initiation than the maintenance of inflammation.

Natural killer cells, which are CD3- and CD56+ represent a class of innate cells and are a large proportion of circulating lymphocytes in humans. One of their functions is thought to be in the killing of cells that lack MHC class I. Given their abundance in humans, it is not surprising that there is a large literature on the role of NK cells in MS, but the literature is large, complex, and frequently contradictory and beyond the scope of this article in reviewing; a recent review of these innate cells and other innate lymphocytes in MS, such as gamma-delta T cells, NKT cells, and innate-like B cells (such as B1 and marginal zone B cells) is helpful (92).

### B Cells and CD8+ T Cells

The most dramatic example of a change from the “antigen-reactive adaptive immunity, particularly CD4+ T cells” dogma for MS pathogenesis was the demonstration that B cells are critical in MS. The MS world was rocked in 2008 by the demonstration of the dramatic efficacy of rituximab, an anti-CD20 monoclonal antibody, in decreasing neuroinflammation in RRMS. The research path by which B cells were demonstrated to be highly effective has been summarized by Steve Hauser in a fascinating recent Charcot Lecture (93). The precise mechanism by which B cell depletion results in decreased CNS neuroinflammation in MS is under active investigation, but remains at this point unclear. There are several plausible hypotheses for why B cells might have a major role in neuroinflammation and CNS damage in MS:

1. antibody secretion- the only cells which make antibodies are B cells, specifically highly differentiated plasma cells, and the presence of plasma cells (82) and antibody production within the CNS (94) in MS is well-established. However, how this local immunoglobulin can contribute to the disease process remains unclear and is reviewed below in the “immunoglobulin” section.

2. Antigen presentation- B cells have been demonstrated to be excellent at antigen presentation and, in line with the “ antigen-reactive adaptive immunity, particularly CD4+ T cells“ dogma, B cells can be hypothesized to be the most prominent antigen presenters to pathogenic T cells, and without that antigen presentation, activation of the pathogenic T cells will not take place (95). Recent work suggests the possibility that self-antigens consisting of HLA-derived self-peptides occur on B cells in MS and can potentially stimulate autoreactive CD4+ T cells (96). These findings, i.e., the importance of HLA-derived self-peptides, require confirmation, and the precise mechanisms involved in the inflammatory process in MS remain conjectural.

3. Production of proinflammatory cytokines and chemokines that propagate inflammation- In an interesting series of experiments, Bar-Or et al. (97) used a dual activation system for B cells by cross-linking the BCR and stimulation through CD40 in the presence of a 3^rd^ signal IFNgamma or the TLR9 ligand CpG DNA. B cells of patients with MS exhibited abnormal lymphotoxin and TNFalpha secretion. B-cell depletion, both *ex vivo* and *in vivo*, using rituximab, resulted in significantly diminished proinflammatory T cell responses, and soluble products from activated B cells of untreated patients with MS reconstituted these diminished T-cell responses. The authors postulated that these results might explain the diminished neuroinflammatory activity in MS as a result of B cell depletion therapy. MS B cells, especially IgD-CD27- cells, produced more TNFalpha and LTalpha than similar B cells from healthy controls (98). However, this area of research has not matured to the point that any B cell cytokine has been targeted for possible treatment (including CXCL13; refer to “cytokine” section below).

4. Production of soluble toxic factors contributing to oligodendrocyte and neuronal injury- Recent research has identified B cell produced non-immunoglobulin “factors” or exosomes (99) as being toxic to both oligodendrocytes (100) and neurons (101). This work is very provocative but needs to be confirmed.

5. Contribution to the formation of ectopic lymphoid follicles (ELFs) in the CNS. In secondary lymphoid organs, such as the lymph nodes and spleen, highly organized areas called germinal centers (102) are the main sites where antigen-activated B-cell clones expand and undergo somatic hypermutation. A subset of T cells, follicular helper T cells (Tfh), are critical for this process, both within germinal centers and ELFs, and their malfunction has been proposed as a potential mechanism for autoimmunity (103). A burgeoning area of research within MS is the study of CNS ELFs, which are aggregates of lymphoid cells within non-lymphoid tissues with varying degrees of similarity to germinal centers within secondary lymphoid organs. ELFs can be found in the CNS in MS (104, 105) and its animal models (47, 106). Some investigators (107), but not others (108), have found that meningeal ELFs are associated with cortical demyelination.

Targeting ELFs may be helpful therapeutically in MS. In a recent study, focal MS-like lesions were induced by injecting heat-killed Mycobacterium tuberculosis into the brains of mice immunized with the encephalitogenic myelin-oligodendrocyte glycoprotein (MOG). Groups of mice that were treated with anti-CD20 antibodies had better outcomes including dissolution of meningeal lymphoid aggregates resembling ELFs (109). Thus, interfering with the formation of ELFs may be one of the mechanisms by which anti-CD20 monoclonal antibodies work in decreasing neuroinflammation in MS.

6. Providing a reservoir for EBV- B cells serve as a reservoir for EBV, and a controversial hypothesis posits that EBV, the most consistent environmental risk factor for MS, latently infects MS subjects' B cells and contributes to MS pathogenesis (110, 111). This hypothesis has been supported by the finding of EBV transcripts by some (112, 113). In contrast, others have not been able to detect EBV in MS CNS (55, 114), and a recent study which probed carefully for EBV in B cells from patients with MS also could not detect any, including in clonally expanded and somatically hypermutated plasmablasts/plasma cells (115).

A type of B cell that has attracted a great deal of interest in MS is the antibody-secreting cell (ASC) since antibody production within the CNS is a prominent feature of MS (see “Immunoglobulin” section below). In humans ASCs consist of highly differentiated B cells, called plasmablasts or plasma cells, depending on how highly differentiated they are. Plasmablasts retain some markers of less differentiated B cells, i.e., CD19, CD20, B-cell receptor for antigen, and have low or absent CD138, while plasma cells are usually CD19neg, CD20neg, BCRneg, CD138 pos. Some investigators feel it is frequently hard to truly differentiate these two types of ASCs, and feel they exist on a continuous spectrum of differentiation (116), and a recent manuscript termed CD19+IgD-CD27hi cells in MS as “plasmablasts/plasma cells” (115). These cells are responsible for the hallmark diagnostic findings in CSF analysis in patients with MS of elevated IgG index and CSF-specific oligoclonal bands.

Although CD4+T cells are indeed found in MS lesions, the predominant type of T cell in all types of brain lesions is CD8+ T cells (37). This predominance is true for all types of MS lesions, be they active, chronic active, and inactive white lesions or normal-appearing white matter; the predominance also holds for studies in patients with various disease durations, tempos of evolution, and therapies. The degree of predominance is large; the CD8:CD4 ratio in the CNS parenchyma of patients with MS averages about 4:1, and its inverse to the CD8:CD4 ratio in the peripheral blood (1:2) and CSF (1:4) of patients with MS and healthy controls (117). Although encephalitogenic T cells in EAE are usually felt to be CD4+, CD8+ T cells can also induce EAE (118, 119). The possible regulatory or encephalitogenic roles of CD8+ T cells have recently been extensively reviewed (120), but this area of research has generally been neglected, and more work needs to be done.

### CNS-Resident Cells

Central nervous system (CNS)-resident cells not normally classified as “immune cells,” also participate in neuroinflammation in MS. Astrocytes, which make up a large percentage of CNS cells, have especially been implicated. However, it is difficult to study this class of cells in the laboratory, and it is known that human astrocytes are very different from rodent astrocytes in that they are over 20-times larger by volume and contact up to 10-times the number of synapses as rodent astrocytes (121), making it tenuous to extrapolate from results from rodent research to humans in this field. Non-immunological functions of astrocytes include contributing to the formation and maintenance of neural circuits by affecting synapse formation, playing a role in energy metabolism, neurotransmitter recycling, among others (122). In MS, their importance has been studied in depth by a group at Montreal Neurological Institute, led by Jack Antel, who have recently summarized this research in a review article (123). Of particular interest is the work of this group, as well as others on the Canadian B Cell Team in MS, on the interaction between astrocytes and B cells, demonstrating that factors from human astrocytes robustly supported human B cell survival, induced B cell upregulation of antigen presentation, and promoted activation of T cells by treated B cells (124). Also of interest is work by Brosnan and Raine (125) who examined astrocytic end-feet carefully in MS lesions, based on the findings on astrocytic end-feet injury in neuromyelitis optica (NMO). They found damage to perivascular astrocyte end-feet early in the disease, as well as hypertrophic astrocytes in adjacent parenchyma. They concluded that their results as well as the literature supported multiple roles for the astrocyte in MS. The ability of astrocytes to have a role in glial scarring may also be important. The role of astrocytes in MS will likely be an area of increasing research in MS, which will be assisted by continued research on astrocytic injury in NMO (126).

## Fiction #5. The Critical Molecules in MS are those Involved in T Cell Function

### FACT #5. A Wide Variety of Molecules Are Important in MS and Most of Them Are Not Involved in T Cell Function

A complete review of molecular mechanisms involved in MS is beyond the scope of this manuscript. The molecules reviewed below have been chosen because of their importance to the practicing neurologist, and for the most part, are molecules detectable in the CSF. Other molecules could also have been selected, although the list below represents molecules that at this point in time seem more important and have attracted significant literature.

### Immunoglobulin

Intrathecal immunoglobulin production is a hallmark of MS (127), and its measurement has been helpful in the diagnosis of MS for decades (21, 128). The biology of this phenomenon is not fully understood. IgG is produced by highly differentiated B cells, ASCs, which are likely recruited into the CNS by intrathecal production of the B-cell active chemokine CXCL13 (48), possibly initially as memory B cells (129); intrathecal production of CXCL13 is described below. The location of immunoglobulin production within the CNS may be in specialized inflammatory areas called ectopic lymphoid follicles (ELFs) (105), first described in 1979 by Prineas (130). These follicles are usually found in the meninges and are associated with more severe disease and cortical pathology (107, 131).

The most prominent and well-studied immunoglobulin isotype in MS is IgG, although other isotypes such as IgM (132, 133) and IgA (134) have also been implicated. Humans have multiple subclasses of IgG (IgG1, IgG2, IgG3, and IgG4), which differ in the constant region, particularly in their hinge and upper CH2 domains; however, no specific subclass specifically has been correlated with MS (135). Two types of measurement of elevated intrathecal production of IgG are commonly used in the diagnosis of MS: OCBs of IgG, and the IgG index. OCBs refer to the presence of IgG within bands when run on gels, usually either acrylamide isoelectric focusing or agarose gels. The precise molecular significance of this phenomenon is not known, but it is assumed to derive from similar isoelectric points of IgG produced by a limited number of ASCs in the CNS, in contrast to the large number of ASCs contributing to the IgG present in serum. The IgG index is in contrast a purely quantitative determination, being the ratio of CSF IgG concentration to CSF albumin concentration divided by the ratio of serum IgG concentration to serum albumin concentration. As the methodologies for OCB determination have been improved over time, it has become the intrathecal IgG production assay of choice, with excellent sensitivity in the diagnosis of MS. In a study of 1,505 Swedish patients with MS, Imrell et al. found positive OCBs in 1,422 (94.5%), a percentage in line with most other studies (5). The positivity rate is so high in MS that “patients who are OCB negative should have their diagnosis closely considered” (94).

The antigenic specificity of intrathecally produced IgG in MS is controversial and has not been conclusively determined. In other neuroinflammatory conditions in which CSF OCBs are determined, the CSF IgG is directed to a large part against the inciting antigen, most conclusively demonstrated in CNS infections, such as the measles virus in subacute sclerosing panencephalitis (136). In MS, a disease in which many have felt that the autoimmune response is directed against CNS myelin, some have found that CSF OCBs appear not to be directed against myelin antigens (137), while others have found that the target of the CNS immune response in some patients with MS is the myelin antigen, myelin oligodendrocyte glycoprotein (138). However, there is no consensus, and studies of the antigenic target(s) have continued (139, 140); a recent study by Prineas and Parratt found no support for the hypothesis that damage to myelin is caused by pathogenic serum anti-myelin or anti-oligodendrocyte autoantibodies (141). In contrast, in a provocative study from the University of Colorado, Blauth et al. (142) examined the binding to CNS tissue of IgG1 monoclonal recombinant antibodies (rAbs) derived from the CSF expanded B cell clones to CNS tissue of patients with MS. Those rAbs that displayed binding to mouse and human CNS tissue bound to antigens preferentially expressed on astrocytes and neurons. Some of the rAbs caused significant myelin loss and astrocyte activation when applied to spinal cord explant cultures in the presence of complement. Their conclusion was that their data implicated intrathecal IgG in MS pathogenesis.

### Complement

Complement is a class of proteins grouped within the innate immune system. The complement system is sometimes referred to as the complement cascade since activation of the initial components frequently leads to a series of events, with multiple downstream effects. The system is best known for “complementing” antibody-mediated killing and lysis of microbes and damaged cells and for initiating inflammation.

The involvement of complement in MS is controversial. In a pioneering study Lucchinetti et al. (10) classified MS pathology into 4 distinct patterns, the most common of which is type II with immunopathological evidence of the deposition of immunoglobulin and activated complement in actively demyelinating lesions. This mode of classification is not universally accepted (11), but the immunostaining of immunoglobulin and complement components in MS lesions is indisputable. Identification of complement involvement on brain biopsies might have therapeutic significance: plasma exchange, which results in decreased complement levels (143), benefitted patients with brain biopsies consistent with type 2 but not type 3 appearance (144, 145). The possible role of complement and complement receptors in MS has been recently reviewed in depth (146, 147).

### Chemokines/Cytokines

The best-documented molecule in MS in this group is CXCL13, a chemokine critical for B cell trafficking and initially called B-lymphocyte chemoattractant (BLC) (148). Involvement of CXCL13 in disease is not unique to MS; it is also highly expressed in Lyme borreliosis (149–151). In MS CXCL13 was first studied in the context of the formation of ELFs in MS (104, 152); CSF CXCL13 was subsequently identified as a potential biomarker in MS (153, 154), or MS treatment (155). A recent study found that intrathecal production of CXCL13, as measured by the CXCL13 index, at the time of the initial clinical demyelinating event in MS, was especially helpful in predicting future neuroinflammatory activity as defined by attacks or new or enhancing MRI lesion (49).

The precise mechanism(s) by which intrathecal production of CXCL13 promotes neuroinflammation is not known. Since CXCL13 is the ligand for the receptor CXCR5, and CXCR5 is primarily expressed by B cells, and since B cell depletion is a powerful therapy to decrease inflammatory events in MS, it is possible that the intrathecal production of CXCL13 functions to increase CNS inflammatory activity solely by recruiting B cells to the CNS. However, CXCR5 is also expressed on follicular helper T cells (Tfh) (156), and recruitment of these cells into the CNS may also contribute. The therapeutic effect of fingolimod, a sphingosine-1-phosphate receptor blocker, in MS, has been attributed to decreasing circulating Tfh cells (157).

### Chitinase

The chitinases are 18 glycosyl hydrolases. Their name derives from their ability to cleave chitin, a natural polysaccharide found in the coating of a variety of pathogens as well as the exoskeleton of insects. 18 glycosyl hydrolases are expressed across a wide range of organisms and are evolutionarily conserved even in mammals that lack chitin. Thus, the role that chitinases play in humans under normal circumstances is unclear (158).

The most well-studied chitinase in MS is chitinase 3-like 1(CHI3l1), also called YKL-40, which is classified as a chi-lectin because it binds chitin but does not have chitinolytic activity. Although not normally expressed at high levels in the CNS, inflammation results in upregulation of expression, especially in reactive astrocytes and infiltrating macrophages. The role of chitinases in MS may not be primarily in inflammation but injury repair. In an interesting series of experiments, Starosssom et al. found that murine chitinases, including CHI3L1, induce oligodendrogenesis, possibly through activation of the epidermal growth factor receptor, and that silencing of the chitinase CHI3L3 worsens the severity of EAE (159). In support of the role of chitinases in injury repair, CSF CHI3L1 levels correlated significantly with concentrations of the CSF axonal damage biomarker, neurofilament light chains (160). CSF CHI3L1 levels have also been found associated with conversion from a first demyelinating event to MS (161, 162).

### Neurofilaments

Neurofilaments are a class of molecules that form the neuronal cytoskeleton, and provide physical stability to axons. They are protein polymers, and normally do not occur at substantial concentrations in the CSF. However, in disease states associated with CNS injury, neurofilaments are released into the CSF and can eventually appear in the serum at low concentrations. Although it has been known for some time that CNS damage is associated with increases in the levels of CSF neurofilaments (163), interest in neurofilaments has escalated recently because of fourth-generation assay methodology, i.e., single-molecule array (SIMOA), that has allowed detection of neurofilaments, primarily neurofilament light (NfL) in the blood (164). Serum Nfl as a biomarker in progressive MS has been recently reviewed (165). In a recently published study of 127 patients with MS, serum Nfl was cross-sectionally associated with walking speed, manual dexterity, and cognitive processing speed, and baseline NfL levels predicted 5-year disability scores. However, r values for these associations were relatively low, and thus the utility for this measurement in individual patients may not be high. In addition, there is a poor inter-laboratory level of agreement. The use of NfL measurement in patients with MS care is an area of active investigation.

### Other Emerging Molecules and Potential Biomarkers

sCD27: It is a protein of about 30 kD and is the soluble, truncated version of the surface molecule CD27. CD27 belongs to the TNF receptor superfamily, is a marker for memory B cells in humans, is found on both B and T cells, and participates in B-cell differentiation and immunoglobulin synthesis. sCD27 is released by activated T and B cells, and its elevated levels in the CSF in MS are consistent with CNS inflammation (166, 167).

Triggering receptor expressed on myeloid cells 2 (TREM2): Triggering receptor expressed on myeloid cells 2 (TREM2) is a cell-surface receptor of the immunoglobulin superfamily that is found in a variety of cell types, but primarily cells of the macrophage/monocyte lineage, including microglia and other tissue macrophages. It is released by these cells in response to activation. This molecule appears to be important in the process of myelin debris clearance; TREM2 knockouts have impaired remyelination after cuprizone treatment (168). CSF TREM2, but not blood TREM2, is elevated in both RRMS and PPMS, but also other inflammatory neurological disease (169), and is correlated with levels of neurofilament and sCD27 (170).

Soluble B cell maturation antigen (sBCMA): BCMA, a member of the TNF-receptor superfamily is preferentially expressed in mature B lymphocytes, and may be important for B cell development. It is considered a survival factor for plasma cells and is increased in the CSF of patients with MS, correlated with intracerebral immunoglobulin production (171). CSF sBCMA levels are higher in untreated than treated patients with MS (172).

## Fiction #6. Neurologists Do Not Need Information from CSF Analysis Anymore Since MRIs and Serum Biomarkers are Now Available; Besides, Lumbar Punctures are Highly Invasive, Associated with a High Risk of Headaches, Old-School and Unnecessary

### FACT #6. CSF Analysis Is a Crucial Component in the Diagnostic Workup in MS, and Possible Adverse Effects Are Rare and Easily Managed When Present

One consequence of the challenge to MS research noted above, i.e., “minimal access to involved tissue,” is the need to find a suitable alternative to the involved tissue. In diseases involving structures outside of the CNS, such as the liver or GI tract, in which there is an unimpeded interchange between the tissue and the circulation, testing of the blood provides clinically useful clues. However, in MS where the blood-brain and a blood-CSF barrier is generally diffusely intact, i.e., CSF/blood albumin quotient is normal in 90% of patients with MS (173), blood is not a good indicator of pathologic events within the CNS. Serum biomarkers, although promising for some molecules such as neurofilament light, need more validation to be clinically useful (174). In contrast, CSF analysis is considered invaluable for the neurologist in evaluating neuro-inflammatory conditions including MS (175).

The high value of the results from CSF analysis has recently been acknowledged by the incorporation of the presence of CSF OCBs in the most recent McDonald criteria for the diagnosis of MS (6), where positive OCBs can fulfill criteria for dissemination in time when dissemination in space in the CNS is present. In addition, in an age when MS misdiagnosis is increasingly common, “OCBs have been shown in several studies to have a high negative predictive value, and thus their absence should be a red flag suggesting the possibility of an alternative diagnosis” (7). CSF analysis for markers other than OCBs is also generally helpful in assisting in the diagnosis of MS.

Neurologists are ordering more and more MRIs of the CNS in the diagnosis and management of MS, and with increasing constraints on clinicians' time, some are relying almost solely on MRIs. However, the presence of white matter lesions has poor specificity for the diagnosis of MS, especially in older patients or those with comorbidities (176). With regard to the issue of invasiveness of LPs or their adverse effects, complications of the procedure are rare, and post-LP headaches can be almost completely prevented by the use of atraumatic needles (177, 178). Thus, the history, exam, and both CSF and MRI results should ideally be used in the workup for possible MS.

## Fiction #7. Relapse Rate, the Endpoint Used by the FDA in the Past to Approve Drugs for MS, Continues to Be Appropriate for Developing More Treatments For MS

### FACT #7. Relapse Rate Has Little to No Correlation With What Patients With MS Want and What Their Treatments Ideally Should Do: i.e., Decrease Disability Accrual Over Time. The Best Endpoint for Future MS Medications Would Be a Disability Endpoint

MS treatments have come a long way since ACTH/corticosteroids were described as being effective 70 years ago (179). No other treatments became available until 1993 when interferon beta-1b was shown to be effective in decreasing relapses in patients with frequent relapses (180). Since that time, 22 more drugs have been approved for MS when pivotal trials have demonstrated that they were able to decrease the frequency of relapses.

Relapse frequency in a 2-year trial was certainly a reasonable primary outcome measure in the 1980's when interferon beta-1b was being tested. At that time, it was thought that the primary driver for disability accrual was relapses. However, that hypothesis has been continually questioned through the years. Confavreux et al. (181) studied 1,844 patients with multiple sclerosis who were followed for a mean of 11 years and concluded that “relapses do not significantly influence the progression of irreversible disability.” These powerful data did not affect the FDA's continued acceptance of relapse reduction as an acceptable primary outcome measure in clinical trials over the subsequent 20 years. More recent data continues to confirm Confavreux's conclusions. Kappos et al. (182) found that “in a typical population with relapsing MS, 80–90% of overall disability accumulation occurred independently of relapses. Together with findings previously obtained in (other studies) our study strongly supports that MS may be a single disease continuum with an underlying progressive disease course and a highly variable superimposed accumulation of disability resulting from relapses with incomplete recovery,” or as concluded in a recent editorial, “a primary progressive disease in all cases, but some patients have superimposed relapses (183).” The term Progression Independent of Relapse Activity (PIRA) or “silent progression” has been used to define this type of disability accrual (182, 184), while disability attributed to relapses has been termed “relapse-associated worsening” (RAW).

Although patients and their providers certainly dislike relapses, their primary concern is decreasing not relapses but accrual of disability. Newly diagnosed patients ask me: “Doc, how long will it be before I am in a wheelchair?” or similar questions related to disability accrual, not “Doc, how much can we decrease relapses?” Given patient preferences and the primary progressive nature of the disease from the outset, why is disability accrual NOT the primary endpoint for new drugs in MS?

One answer to this lies in the approach of companies developing new drugs: pharmaceutical companies insist on short studies, and prefer targets germane to biologies, such as inflammation, that are broadly applicable to other possible diseases. For instance, teriflunomide, one of the therapies for MS, FDA-approved in 2012, is a metabolite of leflunomide (185), a drug commonly used for rheumatoid arthritis for decades. Another example is sphingosine-1-phosphate receptor (S1PR) modulators, of which there are 4 currently FDA-approved in MS (fingolimod, siponimod, ponesimod, and ozanimod); the first drug developed, fingolimod, also called FTY720, was initially meant as an immunosuppressive agent in the treatment of organ transplantation (186). Choosing a target operative in MS (inflammation/immunity) that is also operative in multiple other diseases allows a wide range of opportunities while targeting the mysterious process of CNS injury in MS does not.

Another answer to the question of why disability outcomes are not the primary endpoint for new MS drugs is that measuring disability accrual in MS is extremely difficult, and the optimal mechanisms have not been identified. Thus, a company using disability accrual as a primary outcome would need to go into uncharted waters, not a comfortable venture for pharmaceutical companies. An example of these difficulties is a recent attempt by Biogen to expand to secondary progressive MS the indication of their anti-alpha 4 integrin monoclonal antibody, natalizumab, in the ASCEND study (187), previously only approved for relapsing-remitting MS. ASCEND tested the hypothesis that natalizumab could downregulate the intrathecal inflammatory response in secondary progressive MS (188) and utilized a unique multicomponent primary endpoint for sustained disability progression, the EDSS-plus, comprising the EDSS (189), the 25 foot timed talk, and the 9 hole peg test (190). The EDSS-plus had never previously been used as a primary endpoint in a pharmaceutical trial, and, given the failure for natalizumab to meet the primary endpoint, may never be used again. Thus, the optimal measure to use in trials with disability accrual as a primary endpoint remains unclear, in stark contrast to the 3 decades-plus use of relapses as the primary endpoint.

A third answer to the question of why disability outcomes are not being used as the primary endpoint for new MS drugs is that ameliorating progressive neurological disability has proven to be futile in other neurological diseases. The most aggressively pursued disease in this respect, with the most failed trials, has been Alzheimer's disease (191), and no treatment had been approved from 2003 until 2021. In 2021 drug, aducanumab for Alzheimer's was FDA-approved via the use of the FDA Accelerated Approval Program in which a surrogate endpoint, such as an effect on a biomarker, can substitute for clinical benefit in pivotal trials. In the case of Aducanumab, the surrogate endpoint was decreased beta-amyloid, and the approval came despite lack of significant effect on clinical symptoms. However, this approval was fraught with problems on many levels (192), one of which was whether decreasing beta-amyloid was a reasonable surrogate for clinical effect. Whether this type of approach, i.e., identifying a surrogate endpoint, can be successful for disability accrual in MS, seems unlikely at this point in time.

## Fiction #8. Long-Term Outcomes Have Been Uniformly Improved in Patients With MS by the Institution of Immunosuppressive Drugs (ISDs)

### FACT #8. ISDs May Only Improve Long-Term Outcomes in a Small Subset of Patients Diagnosed With MS Using Current Criteria

MS is a highly heterogeneous disease with respect to long-term outcomes. Some patients with severe MS can progress to bed-bound existence or death within a few years of diagnosis (193) while classical MS plaques can be found in individuals who underwent autopsy after a long symptom-free life, a type of asymptomatic MS (194, 195). Many biologic factors affect long-term outcomes, such as comorbidities and variable potential for CNS plasticity, neuronal repair, and remyelination.

Further complicating the picture are major changes in the past two decades in what is being diagnosed as MS. The spectrum of clinical features in MS as diagnosed in 2021 is VERY different than in 2001. Until 2001, clinicians used the Schumacher or more commonly Poser criteria (3) for the diagnosis which utilized almost exclusively clinical features, primarily attacks. These criteria identified a population with severe MS with 50% of patients requiring aid to ambulate or worse, i.e., EDSS of 6 or greater (189), by 24 years after the onset of MS (196). In 2001, the first iteration of the McDonald criteria was published (197), which for the first time, incorporated MRI criteria, and there were subsequent revisions of the criteria in 2005, 2010, and 2017. Each iteration of the McDonald criteria has resulted in milder and milder presentations being diagnosed as “MS.” Thus, using the 2010 McDonald criteria, Chung et al. (198) found that 42% of patients with MS followed for 30 years had no significant disability (EDSS <3); an equivalent calculation for the Poser criteria was only 14% (196). When such an analysis is performed using the even less rigorous 2017 McDonald criteria, the number of “MS-2017” patients without significant disability 30 years after the diagnosis of MS will likely be >50%.

These changing diagnostic criteria make it essentially impossible to use historical controls (199), especially in the analysis of amelioration of disability accrual. Since placebo controls are now considered unethical in MS, clinical research of future therapies with disability measures as the primary outcome will likely require much more difficulty, compounded by the other problems outlined above in Fiction/Fact #7.

Since the diagnosis of MS is currently being given to a large percentage of people who will not develop a significant disability over the long term, the benefit/cost ratio for ISDs must be carefully evaluated in each patient diagnosed with MS. Of particular relevance to this calculation is a landmark study by Scalfari et al. (200). They found that relapse frequency beyond Year 2 did not predict disability outcomes. They felt that “more frequent attacks during the first 2 years are likely to be concomitant with, rather than causative of, faster disease progression (201),” i.e., that those frequent attacks during the first 2 years did not cause the worse prognosis but rather identified a subset of particularly aggressive MS. Given their data from the study of 28,000 patient-years, the appropriate conclusion would be that, since ISDs predominantly decrease the likelihood of attacks, they may not affect long-term outcomes in almost any patient with MS, and especially not if given more than 2 years after the onset of the disease. Supportive data for the above conclusion comes from a study of the use of interferon beta in 2,656 Vancouver (202). Despite the clear benefit of interferon beta for decreasing relapses, the authors concluded that “among patients with relapsing-remitting MS, administration of interferon beta was not associated with a reduction in progression of disability.”

Given this questionable benefit for long-term outcomes, the cost must be assessed carefully. ISDs significantly increase risks of infection (203), cancer (204, 205), and poor response to vaccines (206). These adverse effects are especially worrisome now during the COVID pandemic (207). In addition, the financial burdens to both patients for out-of-pocket expenses and the health care systems are both onerous and worsening over time (208).

Ideally, a population that would be likely to derive maximal benefit from ISDs could be identified by a biomarker or combination of biomarkers, and this predictive approach could be utilized as soon as possible, i.e., at the time of the initial clinical demyelinating event (ICDE). A highly promising biomarker for prediction of future inflammatory activity (attacks or new lesions on MRI) at the time of the ICDE is intrathecal production of the chemokine CXCL13, as shown recently in a study of 41 patients with ICDE and 26 patients with other forms of MS (49). CXCL13 binds to the chemokine receptor CXCR5 on B cells and follicular helper T cells and serves to chemoattract these cells from the circulation into the CNS. Intrathecal production of the protein is measured by calculating the CXCL13 index [(CSF concentration_CXCL13_/Serum concentration_CXCL13_)/(CSF concentration_albumin_/serum concentration_albumin_)]; neurologists are comfortable with this type of index which is also used for calculation of the IgG index, commonly used in the analysis of CSF in MS. Incorporating serum levels is important since CXC13 is a small molecule that can enter the CNS easily and serum concentrations of CXCL13 in patients with MS are highly variable (209). The albumin ratio is a well-accepted measure of blood-CSF and blood-brain barrier integrity (210), which must be included because decreased integrity will result in higher concentrations coming into the CSF from the serum. We are currently formulating a clinical trial using the CXCL13 index to determine treatment where CXCL13-negative ICDE patients would be treated with moderate efficacy ISDs, such as interferons or glatiramer acetate, and CXCL13-positive ICDE patients would be treated with high efficacy ISDs (211). Of course, ideally using biomarkers for treatment to ameliorate long-term outcomes would be assisted by knowing more about the biology of MS, as noted in a recent review (212) in which the following aspects remain mysterious: when does MS start? does the spectrum of MS really span multiple diseases? when does the progressive phase of the disease begin? in which of the disease is there a therapeutic window for immunotherapy?

Biomarkers other than the CXCL13 index have been utilized to attempt to predict future MS activity in ICDE and other forms of MS. One that has attracted the most excitement in the last few years, both for predicting inflammatory activity and correlating with axonal damage, has been serum neurofilament light, partly because of the easier accessibility of serum relative to CSF, discussed above, and reviewed recently (213). A detailed review of potential biomarkers is beyond the scope of this paper, but there have been several excellent reviews (214, 215).

## Conclusion

This review has sought to clarify some of the controversies surrounding the neuroimmunology of MS, and the relevance of some of the basic aspects of the disease to diagnosis and management. It is not surprising that it is very little about the disease we know with supreme confidence since the immune and nervous systems are the most complex in medicine. There are few other diseases where “personalized medicine” (216) is so important, given the heterogeneity of MS, but our ability to achieve this goal optimally is hindered by our lack of adequate understanding of the disease. However, as our research efforts focus more and more on the disease in humans, and less on relatively unfaithful animal models (15), I am confident that we will reach the goal of understanding enough of the neuroimmunology of the disease so that we can truly personalize therapy to the needs of each patient.

## Author Contributions

The author confirms being the sole contributor of this work and has approved it for publication.

## Funding

This study was funded by Dartmouth-Hitchcock Medical Center, Geisel School of Medicine at Dartmouth, and the Murray B. Bornstein Fund.

## Conflict of Interest

The author declares that the research was conducted in the absence of any commercial or financial relationships that could be construed as a potential conflict of interest.

## Publisher's Note

All claims expressed in this article are solely those of the authors and do not necessarily represent those of their affiliated organizations, or those of the publisher, the editors and the reviewers. Any product that may be evaluated in this article, or claim that may be made by its manufacturer, is not guaranteed or endorsed by the publisher.
